# Author Correction: A systematic genome-wide mapping of oncogenic mutation selection during CRISPR-Cas9 genome editing

**DOI:** 10.1038/s41467-022-30475-5

**Published:** 2022-05-16

**Authors:** Sanju Sinha, Karina Barbosa, Kuoyuan Cheng, Mark D. M. Leiserson, Prashant Jain, Anagha Deshpande, David M. Wilson, Bríd M. Ryan, Ji Luo, Ze’ev A. Ronai, Joo Sang Lee, Aniruddha J. Deshpande, Eytan Ruppin

**Affiliations:** 1 Cancer Data Science Laboratory, Center for Cancer Research, National Cancer Institute, National Institutes of Health, Bethesda, MD 20892 USA; 2 Laboratory of Human Carcinogenesis, Center for Cancer Research, National Cancer Institute, National Institutes of Health, Bethesda, MD 20850 USA; 3 Center for Bioinformatics and Computational Biology, University of Maryland, College Park, MD 20742 USA; 4 Tumor Initiation Program, Cancer Center, Sanford Burnham Prebys Medical Discovery Institute, La Jolla, CA 92037 USA; 5 Laboratory of Molecular Gerontology, National Institute on Aging, Intramural Research Program, National Institutes of Health, Baltimore, MD 21224 USA; 6 Laboratory of Cancer Biology and Genetics, Center for Cancer Research, National Cancer Institute, National Institute of Health, Bethesda, MD 20850 USA; 7 Samsung Medical Center, Sungkyunkwan University School of Medicine, Suwon, 16419 Republic of Korea; 8 Department of Precision Medicine, School of Medicine and Department of Artificial Intelligence, Sungkyunkwan University, Suwon, 16419 Republic of Korea

**Keywords:** Cancer, Computational biology and bioinformatics, CRISPR-Cas9 genome editing

Correction to: *Nature Communications* 10.1038/s41467-021-26788-6, published online 11 November 2021.

The original version of this Article incorrectly acknowledged Eytan Ruppin as the sole corresponding author instead of Aniruddha J. Deshpande and Eytan Ruppin as co-corresponding authors. This has now been corrected in both the PDF and HTML versions of the Article.

