# Supplementary material for: A systematic genome-wide mapping of oncogenic mutation selection during CRISPR-Cas9 genome editing
Source: Nat Commun. 2021 Nov 11;12:6512. doi: 10.1038/s41467-021-26788-6 (PMC8586238; doi:10.1038/s41467-021-26788-6)
Supplement: Supplementary file 1 — Supplementary Information [file 41467_2021_26788_MOESM1_ESM.pdf]

Supplementary Materials for “A systematic genome-wide mapping of oncogenic mutation selection during CRISPR-Cas9 genome editing”

Sanju Sinha[1,2,3,#], Karina Barbosa Guerra[4,#], Kuoyuan Cheng[1,3,#], Mark DM Leiserson[3], Prashant Jain[4], Anagha Deshpande[4], David M Wilson III[5], Bríd M. Ryan[2], Ji Luo[6], Ze'ev A. Ronai[4], Joo Sang Lee[7,*], Aniruddha J. Deshpande[4,*], Eytan Ruppin[1,*]

[1]Cancer Data Science Lab, Center for Cancer Research, National Cancer Institute, National Institute of Health, Bethesda, MD 20892, USA

[2]Laboratory of Human Carcinogenesis, Center for Cancer Research, National Cancer Institute, National Institute of Health, Bethesda, MD 20850, USA

[3]Center for Bioinformatics and Computational Biology, University of Maryland, College Park, MD 20742, USA

[4]Tumor Initiation Program, Cancer Center, Sanford Burnham Prebys Medical Discovery Institute, La Jolla, CA 92037, USA

[5]Laboratory of Molecular Gerontology, National Institute on Aging, Intramural Research Program, National Institutes of Health, Baltimore, MD 21224, USA.

[6]Laboratory of Cancer Biology and Genetics, Center for Cancer Research, National Cancer Institute, National Institute of Health, Bethesda, MD 20850, USA

[7]Samsung Medical Center, Sungkyunkwan University School of Medicine, Suwon 16419, Republic of Korea.

[#]Co-first author

[*]Corresponding author

Supplementary Notes

Table of Contents

1. Testing the effects of potential confounding factors in CDE+/- identification for p53 and KRAS

2. Validations of p53/KRAS in recently published genome wide CRISPR screens from the Sanger Institute

3. CDE+ genes are differentially more essential in primary RPE1 p53 WT vs p53 null across multiple screens from different studies

4. Computation of median mutant selection levels for KRAS and p53

5. Pathways up/downregulated in p53 wildtype vs mutant cell lines in response to Cas9 induction

6. Off-Target DNA damage effect on cell fitness is dependent on CRISPR-selected cancer driver genes

7. VHL as a potential CRISPR-selected cancer driver

8. CRISPR-specific differential essentiality of the top ranked CDE+ gene: TAF8

9. Quality control of publicly available genetic screens used

10. *Genetic screens reveal that CRISPR-KO but not CRISPRi of CDE+ genes induces selection for p53 mutants*

11. *Evidence for selection of mutant p53 based on copy number alterations in CDE+ genes in patients' tumors*

12. *CRISPR-KO of KRAS CDE+ genes induces selection for KRAS mutant cells in KRAS-isogenic cell lines*

13. Re-running the analysis using an expanded version of DepMap

14. Computing mutant selection susceptibility using known essential geneset

1. Testing the effects of potential confounding factors in CDE+/- identification for *p53* and *KRAS*

1a. Functional effect of different p53 variants

Since the functional role of *p53* mutation status is not known, we repeated the identification of CDE genes by *p53* mutation status in CRISPR-Cas9 and shRNA screens, focusing on cell lines harboring known loss-of-function *p53* mutations solely (Methods, N=78). Comparing those with the WT cell-lines as controls (N=75), we observed an even higher significance in the differences of median post-KO/KD cell viability (Chi-squared test P=1.7E-292, **Supplementary Data 2,** Methods; for *KRAS* chi-squared test P=3.1E-92). These findings were further corroborated by a permutation test where the cell line's *p53* mutation status was shuffled 10,000 times (P<1E-4, Methods; for *KRAS* P<1E-04).

1b. Effect of partial vs complete silencing

Considering shRNA knockdown (KD) silences a gene at the mRNA level, this method could potentially result in a partial silencing of a gene activity in contrast to complete removal in case of knockout (KO) via CRISPR-Cas9. This could confound the identification process of CDE genes, in the cases where a gene might be differentially essential only when completely removed. We designed a framework to test the phenomenon of "differentially more killing of p53 or KRAS WT vs their mutant form" independent of this confounding factor. To this end, we repeated our analysis on the genes which are not expressed at all (i.e. read count being 0), and thus would not be affected by this partial vs complete removal phenomenon. We still observed that KO of these non-expressed genes have significantly more deleterious effects on the fitness of *p53* WT compared to mutant cells specifically in CRISPR screens (Wilcoxon Rank Sum P<0.001; for *KRAS* P<4E-09). This can only be attributed to CRISPR-mediated DNA double stranded breaks instead of effects of gene perturbation, since the genes in this analysis were transcriptionally inactive.

1c. Effect of potential functional relationship with p53

Potential functional relationship e.g. synthetic lethal or rescue interaction with p53 may affect our CDE genes identification process. Thus, to test this hypothesis, we repeated the above analysis using non-essential genes mined from Hart et al. 2015. We again observed that KO of these non-essential genes more significantly impair the fitness of *p53* WT compared to mutant cells specifically in CRISPR screens yielding consistent findings (Wilcoxon Rank Sum P<4E-06 ; for KRAS P<8E-05).

2. Validations of *p53/KRAS* in recently published genome wide CRISPR screens from the Sanger Institute

During the process of completion of this manuscript, genome-wide CRISPR-Cas9 screens in 326 cancer cell lines were generated at the Sanger Institute through an independently designed experimental pipeline [1]. Compared to the DepMap, the Sanger Institute's screens include a higher number of sgRNAs per gene and a lower assay dropout length. We mined the shRNA data available for the subset of cell lines used in these screens and repeated the identification of CDE genes and CRISPR-selected cancer drivers (CCDs). First, we indeed identified a high number of DE+ genes (N=752) compared to DE- (N=58), the majority of them being CRISPR-screen specific. In contrast, the shRNA screens again had a balanced number of DE+ and DE- genes (Chi-squared imbalance test P<1.4E-284). We next observed that both CDE+ and CDE- genes identified from both the screens were significantly overlapping (hypergeometric P< 2E-63 and <8E-07, respectively). Next, we repeated the process of CRISPR-selected cancer drivers identification and obtained *KRAS* and *p53* as our two hits with similar and highly overlapping *KRAS* CDE+ and CDE- genesets (hypergeometric P< 2E-108 and <1.3E-19, respectively).

3. CDE+ genes are differentially more essential in primary RPE1 *p53* wild-type vs *p53* null across multiple screens from different studies

We mined seven genome-wide CRISPR-Cas9 KO screens performed in isogenic-p53 cell lines [2] (two p53 mutants, five p53 wildtype). In these screens, we tested whether CDE+ genes KO is differentially more essential in *p53* WT vs mutant cells in a pairwise fashion. We observed that CDE+ genes were differentially less essential in the Hart *et al.* [3] *p53* null screen vs the rest of the *p53* WT screens (P< 2.1E-11 for Zimmerman *et al.* [4], 4.6E-58 for Brown *et al. first* screen [2], 5.5E-80 for Brown *et al. second* screen [2], 2.5E-02 for Hart *et al.* [5], 5.3E-02 for Haapaniemi *et al.* [6]) in contrast to other *p53* null screens (P<5.3E-01, Haapaniemi *et al.* [6]). Similarly, for

the other *p53* null cell lines (Haapaniemi *et al.* [6]), we observed consistent results (P< 1.5E-18 for Zimmerman et al. [4], 3.2E-90 for Brown et al. first screen [2], 5.5E-80 for Brown *et al.* second screen [2], 2.5E-02 for Hart *et al.* [3], 5.3E-02 for Haapaniemi *et al.* [6]).

4. Computation of median mutant selection levels for *KRAS* and *p53*

We calculated a median essentiality difference between the WT and mutant cell lines (for a given CRISPR-selected cancer driver) for each gene CRISPR-KO and took this metric as the CCD mutant selection potential for each gene CRISPR-KO. The median of this score across all the genes is the median *mutant selection level* for a CCD.

5. Pathways up/down-regulated in *p53* WT vs mutant cell lines in response to Cas9 induction

Similar to the analysis performed for *KRAS*, we investigated the pathways differentially regulated by a *p53* mutation upon Cas9 expression (40 *p53* WT and 123 mutant cell lines from [15]) and thus might be involved in the selective advantage of *p53* mutants during CRISPR-KO (**Fig. S9a**). Our top notable pathway hits involved KRAS downregulation, protein secretion, unfolded protein response, interferon gamma response, G2M checkpoint, and DNA repair, in that order. This is in concordance with previous reports [6, 14-16].

6. Off-target DNA damage effect on cell fitness is dependent on CRISPR-selected cancer driver genes

Based on the notion that the CCDs could regulate the DNA damage response induced by Cas9, we hypothesize that the level of sgRNA off-target effect is associated with the extent of the DNA damage response and consequently cell viability after gene knockout (KO), and that this is dependent on the mutation status of CCDs. Specifically, the level of sgRNA off-target effect will be negatively correlated with post-KO cell viability, but preferentially only in the CCD-wildtype cells. Contribution of off-target to cell fitness is a combined effect of DNA damage and silencing of off-target genes. To avoid confounding by the latter, we considered only potential off-target hits in the non-coding regions and calculated an off-target score (0 to 1, where 0 represents no off-target hits) for each sgRNA by taking into account both sgRNA mismatch position and mismatch type formulated by Doench et al. 2016. Specifically, for each sgRNA sequence, we calculated the genome-wide off-target score and list of potential off-target sites using CRISPRseek [*7]*. The top

100 potential off-target sites were taken into consideration to compute an overall cut-off frequency for the determination of an off-target score [8]. We observed a significantly stronger positive correlation between gene essentiality and off-target score in CCD-wildtype vs mutant cell lines ($P<0.01$ for *p53* and $P<0.01$ for *KRAS*). This difference in correlation strength further increases if we only consider top genes ranked by off-target score inducing a higher extent of DNA damage. These results suggest that similar to *p53*, the CDE effects of *KRAS* are also likely mediated by their potential role in the DNA damage response.

Based on the above computed off-target score, we took the *x* top ranked genes with the highest off-target scores and tested for their enrichment for the *CDE+* genes for each CCD, where the value of *x* is the number of the respective *CDE+* genes. The fraction of CDE+ genes that were also among the top *x* genes with the highest off-target scores (for *KRAS* and *p53*) was used as a measure of the accountability of CDE+ genes by sgRNA off-target effects. We repeated this analysis in different settings to test the robustness of these results (considering top 50 and top 200 potential off-target sites, and only taking into account mismatch position to calculate off-target score) and observed concordant extent of enrichment.

7. *VHL* as a potential CRISPR-selected cancer driver

Based on the count of CDE+ genes (**Fig. 5a**), the third ranked CCD is *VHL*, where the absolute number of its CDE+ genes is smaller than that of *p53*, the ratio of its CDE+ to CDE- genes is strikingly higher than that observed for *p53*, while the parallel distribution observed in the shRNA-KD screens is balanced. Just like *p53*, the mutational status of *VHL* is significantly associated with the essentiality of CDE+ genes independent of copy number. The CDE+ genes of *VHL* were also enriched in chromosomal bands of CFSs (hypergeometric $P<2.4e-2$). Indeed, *VHL* can act as a positive regulator of *p53* in DNA damage-induced cell cycle arrest or apoptosis [11], possibly accounting for its role as a CCD.

8. CRISPR-specific differential essentiality of the top ranked CDE+ gene: *TAF8*

We show the distribution of cell-lines' *p53* status and copy number with the cell-lines ordered by cell viability after CRISPR-KO of the top CDE+ gene, *TAF8,* as an example (**Supplementary Figure 11**).

9. Quality control of publicly available genetic screens used

For each published genetic screen used in this study, we quantified and used a quality metric to make sure we only analyze high quality screens. To this end, we first obtained gold-standard essential and non-essential genesets from Hart *et al.* [3]. To test the quality of each genetic screen we computed an area under the receiving operator curve (AUROC) using the average logFC across replicates (**Fig. S10**). In this study, we only considered the genetic screens with an AUROC>0.6 (random model AUROC=0.5).

10. Genetic screens reveal that CRISPR-KO but not CRISPRi of CDE+ genes induces selection for *p53* mutants

Given that some of these published screens [1-6] used only few sgRNAs per gene, we performed our own CRISPR screens in a pair of isogenic *p53* WT and *p53* R248Q mutant MOLM13 leukemia cell lines, with a focused sgRNA library targeting top p53 CDE+ and, CDE-, and non-CDE+/- genes (involving 10 guides per gene, Methods). As a control to ensure that the CDE selection effects were specific to CRISPR-KO, the same genes were also targeted by a pooled CRISPR-interference (CRISPRi) library using a catalytically inactive Cas9, fused to the KRAB repressor and the methyl CpG binding protein MeCP2 [9] (illustrated in **Fig. 2a**). Since our initial comparison of CDE essentiality was conducted between the CRISPR-KO and shRNA screens, we set to eliminate inherent differences between CRISPR-Cas9 and shRNA-based approaches by performing the CRISPR-KO and CRISPRi of the same genes with equal number of sgRNAs in an isogenic setting. Similar to the published screens, we used our quality control test to discard any low-quality replicates. In these screens, we identified the genes showing higher essentiality in *p53*-WT vs *p53* mutant cells, but only in CRISPR-KO and not CRISPRi. We confirmed that these genes were enriched for the *p53* CDE+ genes identified earlier in the overall analysis (hypergeometric P<1E-8, Methods). A parallel enrichment of the predicted CDE- genes was confirmed using a similar approach (hypergeometric P<2E-4). We observed that the CDE+ genes were differentially more essential in WT than mutants, specifically in CRISPR-Cas9 KO but not in CRISPRi screens (Wilcoxon P<1E-06 for CRISPR-KO, P<0.32 for CRISPRi). Such a trend of differential essentiality was also observed for the top 10% of CDE+/- genes and is depicted in **Fig. 2b** (Methods).

11. Evidence for selection of mutant *p53* based on copy number alterations in CDE+ genes in patients' tumors

A. *p53*: Given that the CRISPR-KO of CDE+ genes preferentially reduces the viability of *p53* WT cells, we hypothesized that somatic copy number alterations in CDE+ genes (which could act as a possible surrogate for number of DSBs) could also reduce the fitness of *p53* WT tumors. To test this hypothesis, we analyzed the absolute magnitude of somatic copy number alteration (SCNA, taking into account both amplifications and deletion events, see Methods) and patient survival data of 7,547 samples from The Cancer Genome Atlas (TCGA) [10]. As a control, we used genes whose essentiality is not associated with *p53* mutational status (Methods). We found that the absolute SCNAs of CDE+ genes were significantly lower compared to those of control genes in *p53* WT but not *p53* mutant tumors (Wilcoxon rank sum P=7.1E-29, effect size -0.13 for CDE+ vs P=0.13 and effect size -0.017 for the control). This suggests that copy number variations in CDE+ genes were selected against specifically in *p53* WT tumors, as *p53*-mediated responses are detrimental to their fitness. In addition, we observed that CDE+ genes, but not the control genes, were enriched for genes whose high absolute SCNA were associated with accelerated accumulation of *p53* mutation with tumor state (Fisher's test P=0.004, odds ratio=1.84 for CDE+ vs P=0.96, odds ratio=0.62 for the controls; Methods), further supporting the notion that copy number alterations specifically in the CDE+ genes can drive the selection for *p53* mutant tumors. Taken together, these observations provide further evidence for *p53* mutant selection in patients' tumors via the copy number changes in CDE+ genes.

B. *KRAS*: Extending *KRAS* mutant selection *in vitro* results, to the study of SCNA of *KRAS* CDE+ genes in the TCGA tumor patients, we also found that the *KRAS* CDE+ genes, but not the control genes (i.e. those not showing differential essentiality, Methods), were enriched for genes whose high absolute SCNA were associated with accelerated accumulation of *KRAS* mutation with tumor state (Fisher's test P=0.002, odds ratio=1.59 for CDE+ vs P=0.46, odds ratio=1.03 for the controls; Methods), suggesting that SCNA in the *KRAS* CDE+ genes can drive *KRAS* mutations in tumors.

12. CRISPR-KO of *KRAS* CDE+ genes induces selection for *KRAS* mutant cells in isogenic setting

Next, similar to our experiments on *p53*, we generated a focused in-depth sgRNA library consisting of 10 sgRNAs for each of the top 186 *KRAS* CDE+/- genes and performed both CRIPSR-Cas9 and CRISPRi screen in a pair of WT and isogenic *KRAS* G12D mutant MOLM13

cell lines (Methods). We first confirmed that *KRAS* CDE+/CDE- genes are differentially more/less essential in wildtype than in mutant in CRISPR-Cas9 screens (Wilcoxon paired P=0.074 and 0.042 for CDE+ and CDE- genes, respectively) but not in CRISPRi screens (Wilcoxon paired P=0.22 and 0.49 for CDE+/- genes, respectively, **Fig. 6a**). We further confirmed that CDE+/- genes derived from this experiment were highly enriched in our previously identified *KRAS* CDE+/- genes from pooled screens (Methods; hypergeometric P=0.002 for CDE+ and P=0.006 for CDE-). Similar results were obtained from analyzing published genome-wide CRISPR-Cas9 [12] and shRNA genetic screens [13] performed in a different pair of *KRAS* isogenic cell lines (WT and G13D mutation in DLD1 cell line; **Fig. 6b**). Extending these *in vitro* results to the study of SCNA of *KRAS* CDE+ genes in the TCGA tumor patients, we also found that the *KRAS* CDE+ genes, but not the control genes (i.e. those not showing differential essentiality, Methods), were enriched for genes whose high absolute SCNA was associated with accelerated accumulation of *KRAS* mutation with tumor state (Fisher's test P=0.002, odds ratio=1.59 for CDE+ vs P=0.46, odds ratio=1.03 for the controls; Methods), suggesting that SCNA in the *KRAS* CDE+ genes can drive *KRAS* mutations in tumors.

13. Re-running the analysis using an expanded version of DepMap

We mined the latest version of DepMap comprising 521 cell lines with both CRISPR-KO and shRNA-KD screens and repeated the identification of CDE genes and CCDs. Consistent with our previous findings, we observed a high number of DE+ genes (N=910) compared to DE- (N=31), the majority of them being CRISPR-screen specific. In contrast, the shRNA screens again had a balanced number of DE+ and DE- genes (Chi-squared imbalance test P<3.8E-201). We next observed that both CDE+ and CDE- genes identified from both the screens were significantly overlapping (J-index=0.78 and 0.24 for CDE+ and CDE-, respectively, hypergeometric P< 2E-104 and <3.2E-15, respectively). Next, we repeated the process of CRISPR-selected cancer drivers (CCD) identification and obtained *KRAS* and *p53* as our two top hits.

14. Computing mutant selection susceptibility using known essential geneset

We also computed the mutant selection susceptibility for each driver gene using the mean difference between genes essentiality of known essential genes (Ribosomal genes) in WT vs mutant cell lines in the CRISPR-Cas9 screen, vs this score in the shRNA screen. To overcome

screen-specific variance, we centered and standardized the standard deviation of both datasets. To identify drivers whose selection is specific to CRISPR-KO, we looked for drivers whose susceptibility scores are very high in the CRISPR-Cas9 screen but low in shRNA-screen (**Supplementary Figure 13**). Our top hits ranked by high susceptibility scores in the CRISPR-Cas9 screen but low in shRNA-screen (rank difference) are RB1, FOXL2 and CSF1R (Top three hits in respective order, **Supplementary Figure 13**). Here, KRAS and p53 were ranked 7th and 11th, respectively. We note that the essential gene identity was derived from shRNA screens and thus this analysis may have confounding factors.

Supplementary Figures

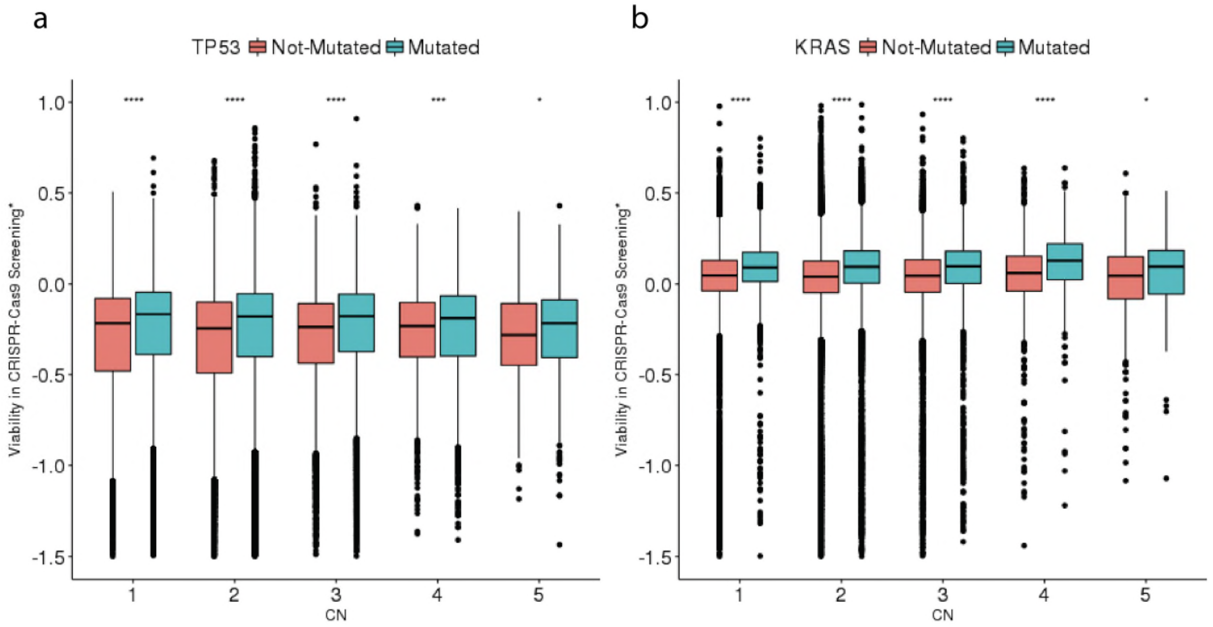

Supplementary Figure 1. **The effect of CRISPR-selected cancer drivers (CCDs) is independent of gene copy number**. The x-axis shows the copy number and the y-axis shows the cell viability after CRISPR-KO of each gene. Red bars denote the cell viability in the cell lines where the CCD is WT, and green bars denote that where the CCD is mutated for (a) *TP53* and (b) *KRAS*. CN=1 denotes cases with copy number less than or equal to 1 and CN=5 denotes cases with copy number greater than or equal to 5. The number of stars at the top of the boxplot represents the significance which is calculated using a one-sided Wilcoxon rank sum test of the difference. *P<0.05, **P<0.01, ***P<0.001, ****P<0.0001. In (a), the exact P values from left to right are 7.0E-06, 9.2E-05, 1.8E-05, 7.2E-03, 8.3E-02. In (b), the exact P values from left to right are 9.4E-06, 4.1E-05, 3.9E-05, 5.3E-05, 6.2E-02. In the boxplots, the center line, box edges and whiskers denote the median, interquartile range and the rest of the distribution in respective order, except for points that were determined to be outliers using a method that is a function of the interquartile range, as done for standard box plots. In both (a) and (b), N=15718 genes (total data points, biological replicates) were used to derive the statistics.

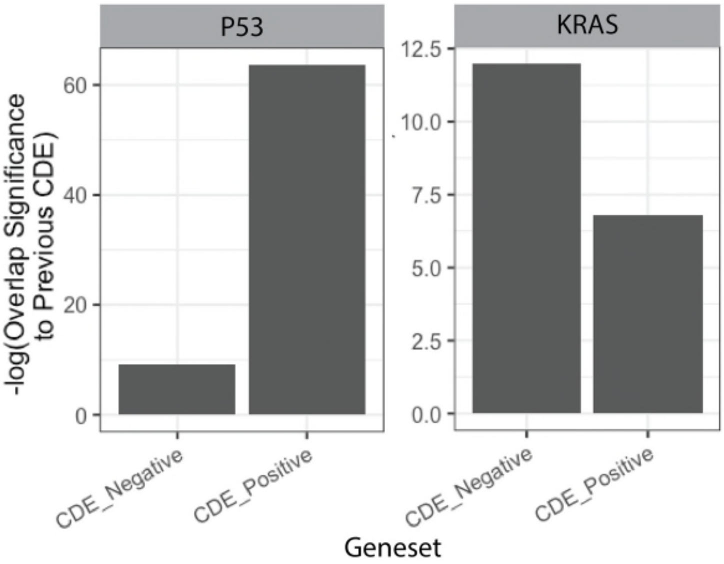

Supplementary Figure 2: Independent support from large-scale CRISRP-Cas9 screen from Sanger institute. Overlap significance (y-axis) between CDE+/- (x-axis) genes identified from DepMap and Sanger screens for **a)** p53 and **b)** KRAS. P values are calculated using one-tailed hypergeometric tests.

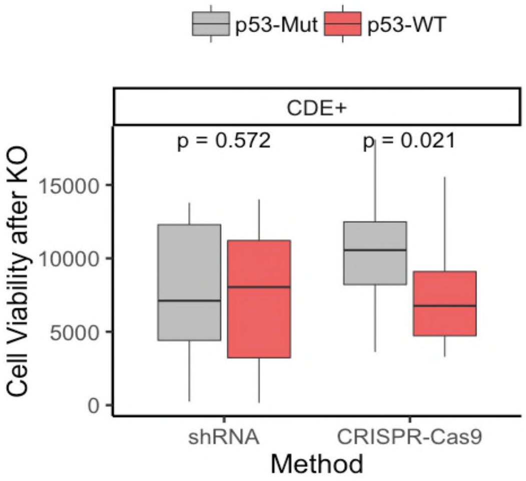

Supplementary Figure 3: Validations of *p53* CDE genes in published CRISPR screens in *p53*-isogenic cell lines. Genome wide published CRISPR-Cas9 and shRNA screens were mined where the top CDE genes knockouts were performed in isogenic WT and mutant cell lines for *p53*. The box plot shows that the sgRNAs of the CDE+ genes become significantly more depleted in WT cells vs mutant cells. The P value of one-tailed Wilcoxon signed-rank test is shown. The p-values were calculated using two-sided Wilcoxon Rank Sum tests. In the boxplots, the center line, box edges and whiskers denote the median, interquartile range and the rest of the distribution in respective order, except for points that were determined to be outliers using a method that is a function of the interquartile range, as done for standard box plots. N=861 CDE+ genes (total data points, biological replicates) used to derive the statistics.

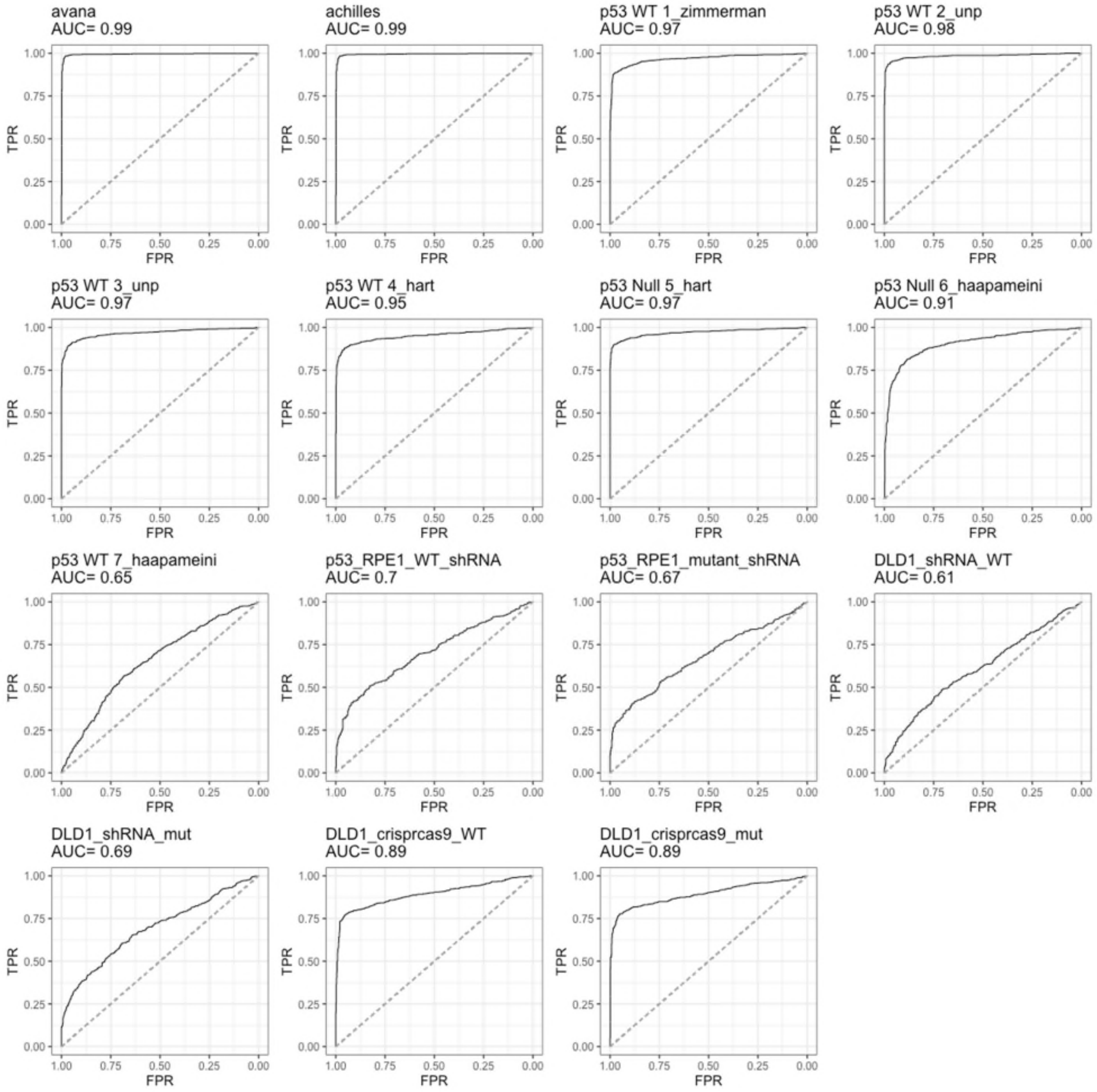

Supplementary Figure 4: Quality metric of published screens: For each published screen used in this study, we computed the area under the receiving operating curve (AUROC) as a predictive power to distinguish between essential and non-essential genes (Hart et al. [46]) as a quality metric. We computed this for the following fifteen screens (panels in order) - **A.** DepMap CRISPR-Cas9 screen (median across cell lines, *AVANA*), **B.** DepMap shRNA screen (median across all the cell lines, *Achilles*), **C-I.** CRISPR-Cas9 screens in RPE1 (p53 WT vs mutant) cells from Zimmerman et al 2018 (zimmerman), Brown et al 2019 (unp), Hart et al. 2015 (hart), Haapaniemi et al. 2018 (Haapaniemi), **J-K.** shRNA screen in RPE1 (p53 WT vs mutant) from Sokolova et al. 2017, **L-O.**

CRISPR-Cas9 and shRNA screen in DLD1 (KRAS WT vs mutant) from Sokolova et al. 2017. Each panel provides the respective ROC curve for the screen providing a trade-off between false positive rate (FPR) and true positive rate (TPR). Area under the diagonal dotted line segment denotes AUROC from a random curve (0.5). The ROC is computed using the pROC R package.

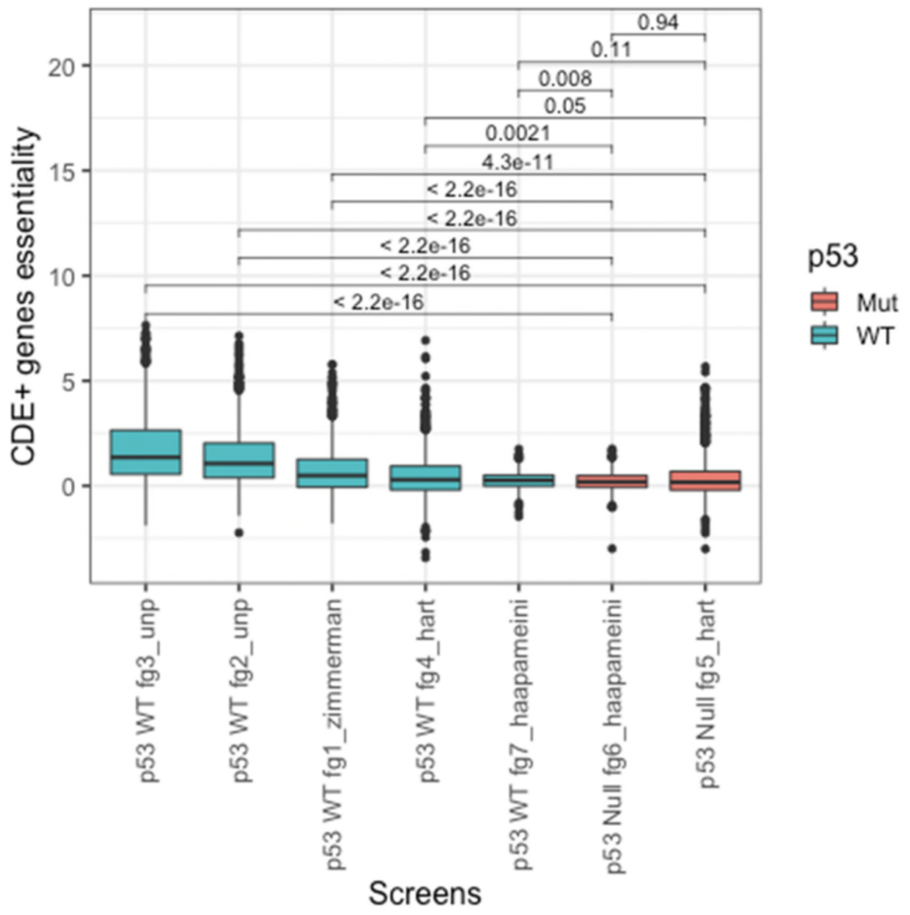

Supplementary Figure 5: Comparison of CDE+ genes CRISPR-Cas9 essentiality across p53 wildtype vs mutant primary RPE1 cell lines curated by Brown et al. [2]. This dataset comprises genome-wide CRISPR-Cas9 screens from five studies - Brown et al 2019 (fg2/3_unp), Zimmerman et al 2018 (fg1_zimmerman), Hart et al. 2015 (fg3/4_hart), Haapaniemi et al. 2018 (fg7/6_Haapaniemi). 2/7 screens were performed on p53 mutant/null and the rest were performed on p53 WT RPE1 cells. The p-values were calculated using two-sided Wilcoxon Rank Sum tests. In the boxplots, the center line, box edges and whiskers denote the median, interquartile range and the rest of the distribution in respective order, except for points that were determined to be outliers using a method that is a function of the interquartile range, as done for standard box plots. N=861 CDE+ genes (total data points, biological replicates) used to derive the statistics. In this plot, the exact p-values for the comparisons for which only the upper bound of the p-values are provided in the plot (From left to right), are 9.9E-28, 3.9E-25, 3.4E-23, 1.5E-20, 4.6E-17.

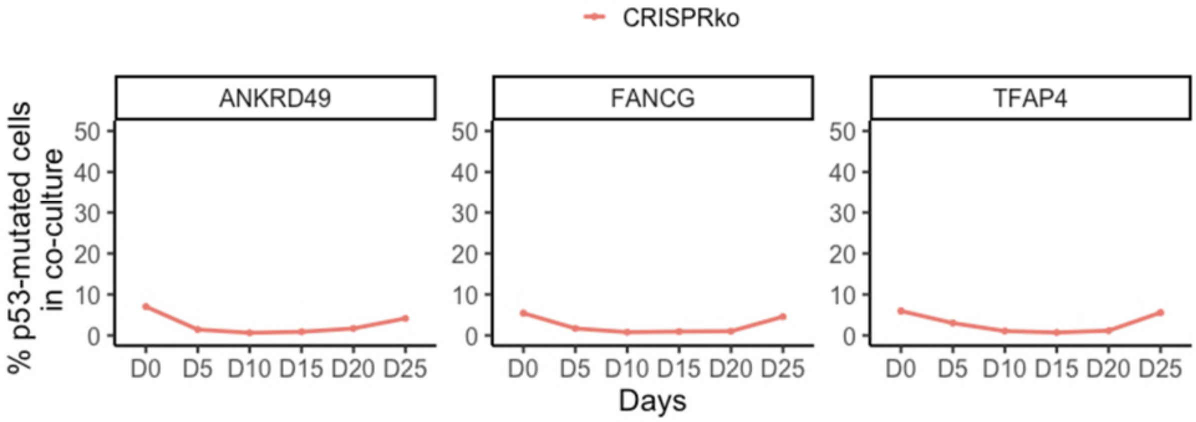

Supplementary Figure 6: Three CDE+ genes out of five showing no selection for *p53* mutant cells under CRISPR-Cas9 knockout of in a co-culture of *p53* WT/mutant cells. In the competition assay, where isogenic *p53* WT/mutant MOLM13 cell lines were mixed with a ratio of 5:95 and top *p53* CDE+ genes were knocked out by CRISPR-Cas9. Change in the percentage of *p53* mutant cells in the p53 mutant-WT cells (Y-axis) co-culture with time (X-axis, number of days in co-culture), under the CRISPR-KO of individual selected top p53 CDE+ genes targeting sgRNA. The p-values are calculated using two-sided Wilcoxon Rank Sum tests. Here, the y-axis scale is kept consistent to the main text.

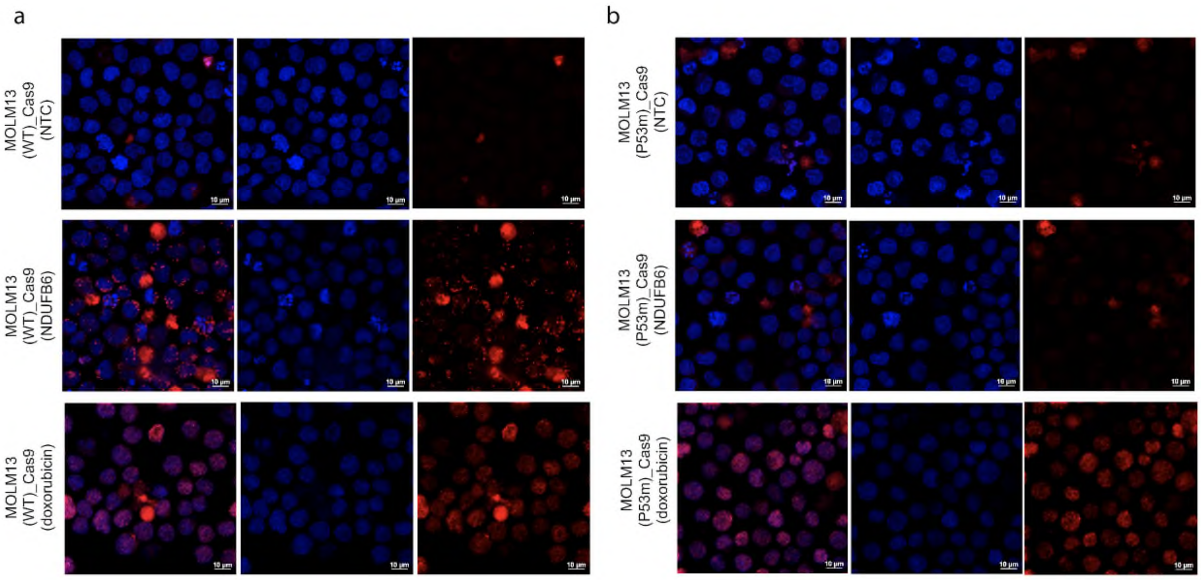

Supplementary Figure 7: Effect of lentiviral delivered Cas9+sgRNA targeting CDE+ gene on DNA damage: DNA damage in Isogenic P53 **(a)** wildtype MOLM13 cells vs **(b)** MOLM13-P53 mutant cells is shown by gH2AX staining with a non-targeting control (top panel), an NDUFB6 sgRNA (middle panel) or with Doxorubicin as a positive control for DNA damage. Images of the cell nucleus marked with DAPI (blue), gH2AX (red) or overlay are shown. This experiment was repeated thrice.

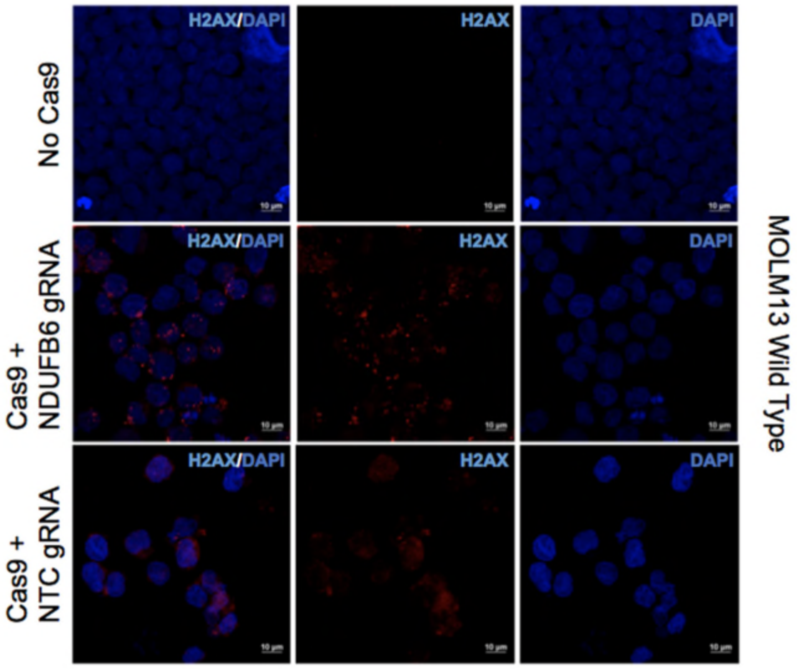

Supplementary Figure 8: Effect of RNP delivered Cas9+ sgRNA targeting CDE+ gene on DNA damage: DNA damage is shown as measured by gH2AX staining in p53 WT MOLM13 cells with no Cas9, Cas9 + an NDUFB6 sgRNA, or a non-targeting control (NTC). gH2AX foci in all three conditions are enumerated in the violin plot in the right panel. This experiment was repeated thrice.

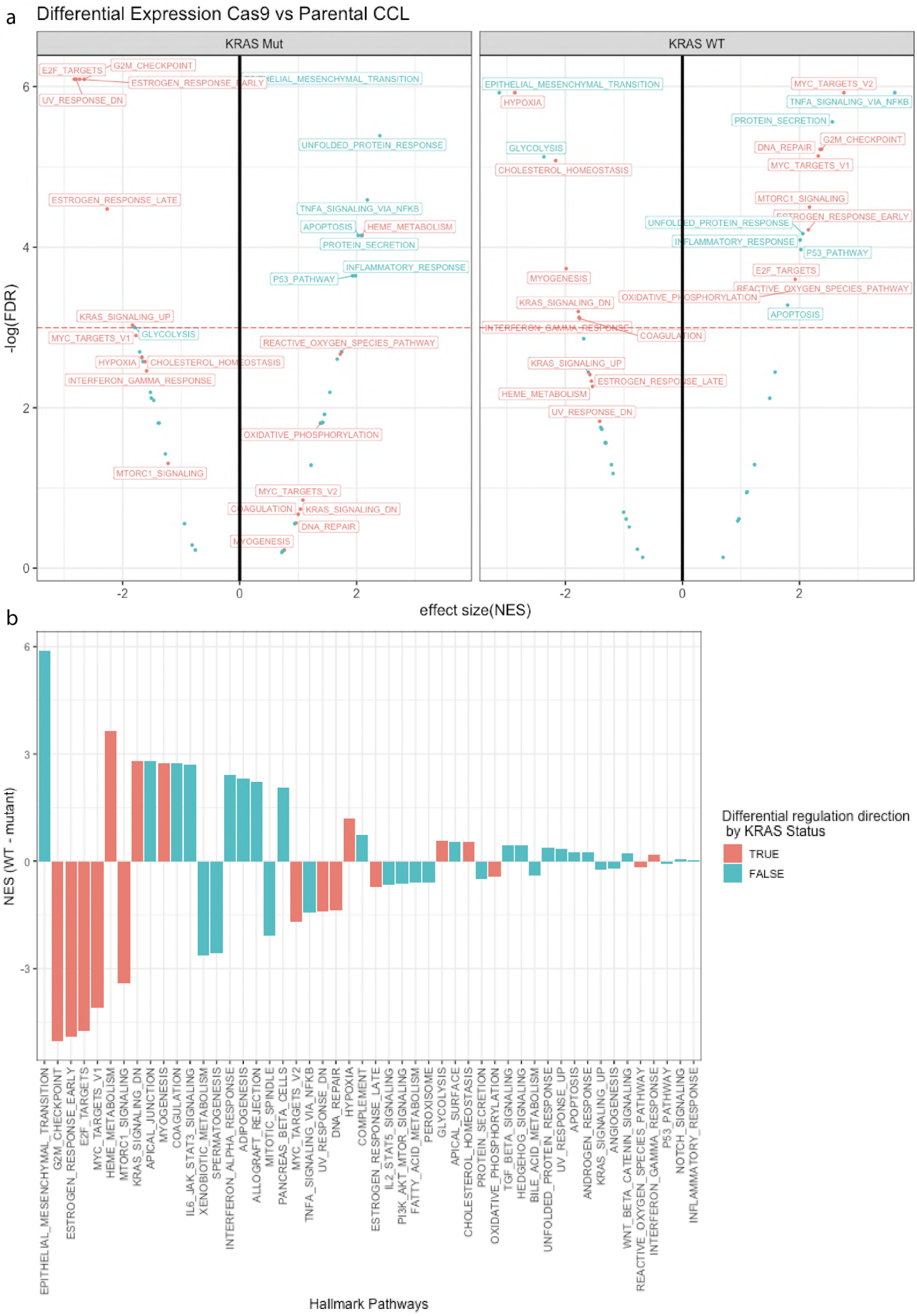
a
Differential Expression Cas9 vs Parental CCL
KRAS Mut
KRAS WT
-log(FDR)
effect size(NES)
E2F_TARGETS
G2M_CHECKPOINT
ESTROGEN_RESPONSE_EARLY
UV_RESPONSE_DN
UNFOLDED_PROTEIN_RESPONSE
ESTROGEN_RESPONSE_LATE
TNFA_SIGNALING_VIA_NFKB
APOPTOSIS
HEME_METABOLISM
PROTEIN_SECRETION
INFLAMMATORY_RESPONSE
P53_PATHWAY
KRAS_SIGNALING_UP
MYC_TARGETS_V1
GLYCOLYSIS
REACTIVE_OXYGEN_SPECIES_PATHWAY
HYPOXIA
CHOLESTEROL_HOMEOSTASIS
INTERFERON_GAMMA_RESPONSE
OXIDATIVE_PHOSPHORYLATION
MTORC1_SIGNALING
MYC_TARGETS_V2
COAGULATION
KRAS_SIGNALING_DN
DNA_REPAIR
MYOGENESIS
EPITHELIAL_MESENCHYMAL_TRANSITION
MYOGENESIS
KRAS_SIGNALING_DN
E2F_TARGETS
ESTROGEN_RESPONSE_LATE
b
NES (WT - mutant)
Hallmark Pathways
Differential regulation direction by KRAS Status
TRUE
FALSE
EPITHELIAL_MESENCHYMAL_TRANSITION
G2M_CHECKPOINT
ESTROGEN_RESPONSE_EARLY
E2F_TARGETS
MYC_TARGETS_V1
HEME_METABOLISM
MTORC1_SIGNALING
KRAS_SIGNALING_DN
APICAL_JUNCTION
MYOGENESIS
COAGULATION
IL6_JAK_STAT3_SIGNALING
XENOBIOTIC_METABOLISM
SPERMATOGENESIS
INTERFERON_ALPHA_RESPONSE
ADIPOGENESIS
ALLOGRAFT_REJECTION
MITOTIC_SPINDLE
PANCREAS_BETA_CELLS
MYC_TARGETS_V2
TNFA_SIGNALING_VIA_NFKB
UV_RESPONSE_DN
DNA_REPAIR
HYPOXIA
COMPLEMENT
ESTROGEN_RESPONSE_LATE
IL2_STAT5_SIGNALING
PI3K_AKT_MTOR_SIGNALING
FATTY_ACID_METABOLISM
PEROXISOME
GLYCOLYSIS
APICAL_SURFACE
CHOLESTEROL_HOMEOSTASIS
PROTEIN_SECRETION
OXIDATIVE_PHOSPHORYLATION
TGF_BETA_SIGNALING
HEDGEHOG_SIGNALING
BILE_ACID_METABOLISM
UNFOLDED_PROTEIN_RESPONSE
UV_RESPONSE_UP
APOPTOSIS
ANDROGEN_RESPONSE
KRAS_SIGNALING_UP
ANGIOGENESIS
WNT_BETA_CATENIN_SIGNALING
REACTIVE_OXYGEN_SPECIES_PATHWAY
INTERFERON_GAMMA_RESPONSE
P53_PATHWAY
NOTCH_SIGNALING
INFLAMMATORY_RESPONSE

Supplementary Figure 9: Pathways up/downregulated in KRAS wildtype vs mutant cell lines in response to Cas9 induction. (a) Top enriched hallmark pathways from MSigDB were tested for enrichment using GSEA in differential expressed genes in parental vs Cas9-induced cancer cell lines with KRAS WT (left panel) and mutant (right panel)**.** The normalized enrichment score (x-axis) and significance for enrichment is provided for each pathway. **(b)** For each hallmark pathway, the difference in NES score in WT and mutant cancer cell lines (x-axis) is provided. Pathways which were significantly up/down regulated in either KRAS WT or mutant are labelled. The pathways whose direction changes by KRAS status are colored orange and the rest are green. Significance was calculated using the GSEA method as implemented in the R package fgsea (Sergushichev et al. 2016).

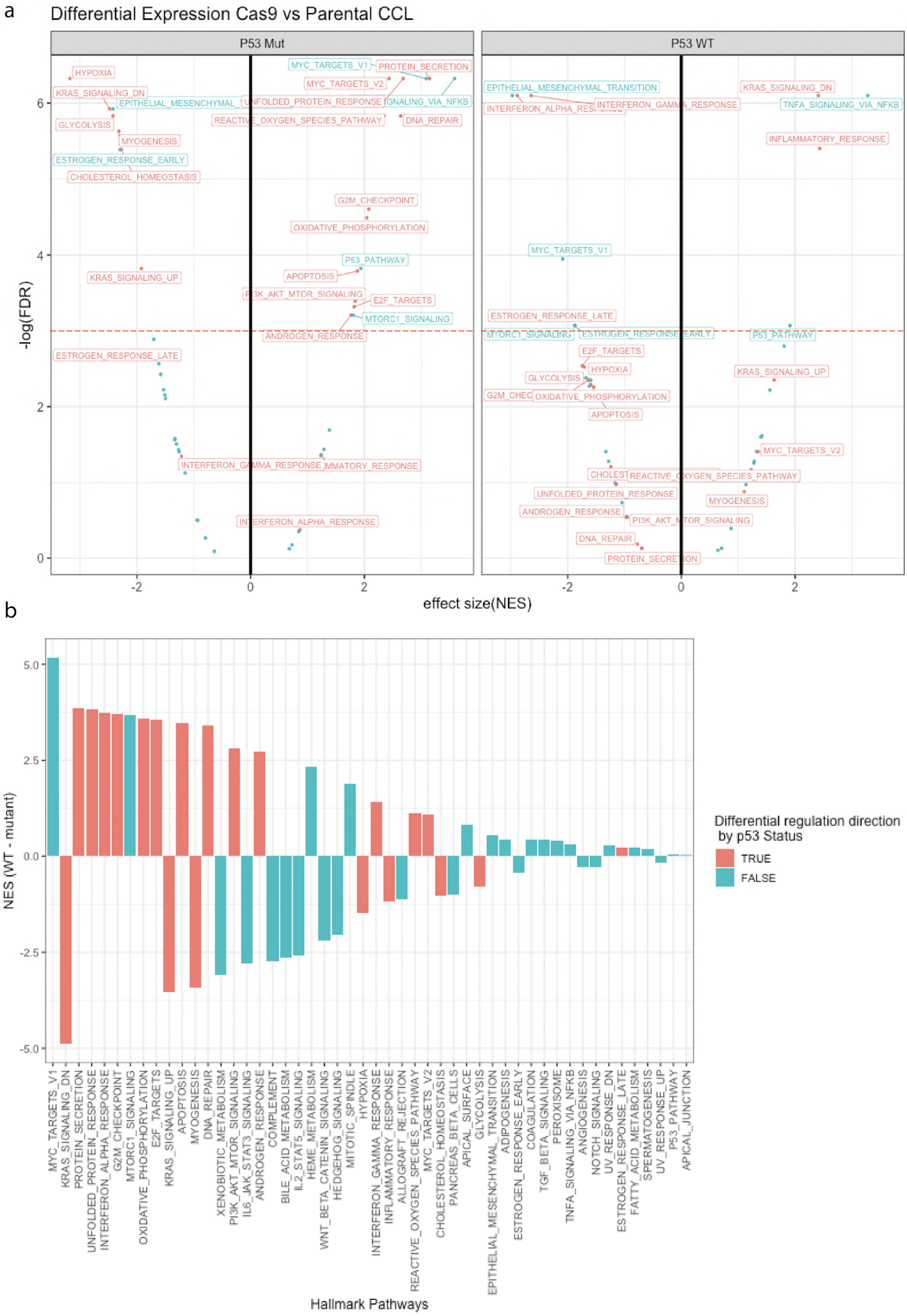
a
Differential Expression Cas9 vs Parental CCL
P53 Mut
P53 WT
-log(FDR)
effect size(NES)
b
NES (WT - mutant)
Hallmark Pathways
Differential regulation direction
by p53 Status
TRUE
FALSE
MYC_TARGETS_V1
KRAS_SIGNALING_DN
PROTEIN_SECRETION
UNFOLDED_PROTEIN_RESPONSE
INTERFERON_ALPHA_RESPONSE
G2M_CHECKPOINT
MTORC1_SIGNALING
OXIDATIVE_PHOSPHORYLATION
E2F_TARGETS
KRAS_SIGNALING_UP
APOPTOSIS
MYOGENESIS
DNA_REPAIR
XENOBIOTIC_METABOLISM
PI3K_AKT_MTOR_SIGNALING
IL6_JAK_STAT3_SIGNALING
ANDROGEN_RESPONSE
COMPLEMENT
BILE_ACID_METABOLISM
IL2_STAT5_SIGNALING
HEME_METABOLISM
WNT_BETA_CATENIN_SIGNALING
HEDGEHOG_SIGNALING
MITOTIC_SPINDLE
HYPOXIA
INTERFERON_GAMMA_RESPONSE
INFLAMMATORY_RESPONSE
ALLOGRAFT_REJECTION
REACTIVE_OXYGEN_SPECIES_PATHWAY
MYC_TARGETS_V2
CHOLESTEROL_HOMEOSTASIS
PANCREAS_BETA_CELLS
APICAL_SURFACE
GLYCOLYSIS
EPITHELIAL_MESENCHYMAL_TRANSITION
ADIPOGENESIS
ESTROGEN_RESPONSE_EARLY
COAGULATION
TGF_BETA_SIGNALING
PEROXISOME
TNFA_SIGNALING_VIA_NFKB
ANGIOGENESIS
NOTCH_SIGNALING
UV_RESPONSE_DN
ESTROGEN_RESPONSE_LATE
FATTY_ACID_METABOLISM
SPERMATOGENESIS
UV_RESPONSE_UP
P53_PATHWAY
APICAL_JUNCTION

Supplementary Figure 10: Pathways up/downregulated in *p53* wildtype vs mutant cell lines in response to Cas9 induction. (a) Top enriched hallmark pathways from MSigDB were tested for enrichment using GSEA in differential expressed genes in parental vs Cas9-induced cancer cell lines with *p53* WT (left panel) and mutant (right panel)**.** The normalized enrichment score (x-axis) and significance for enrichment is provided for each pathway. **(b)** For each hallmark pathway, the difference in NES score in WT and mutant cancer cell lines (x-axis) is provided. Pathways which were significantly up/down regulated in either *p53* WT or mutant are labelled. The pathways whose direction changes by *p53* status are colored orange and the rest are green. Significance was calculated using the GSEA method as implemented in the R package fgsea (Sergushichev 2016).

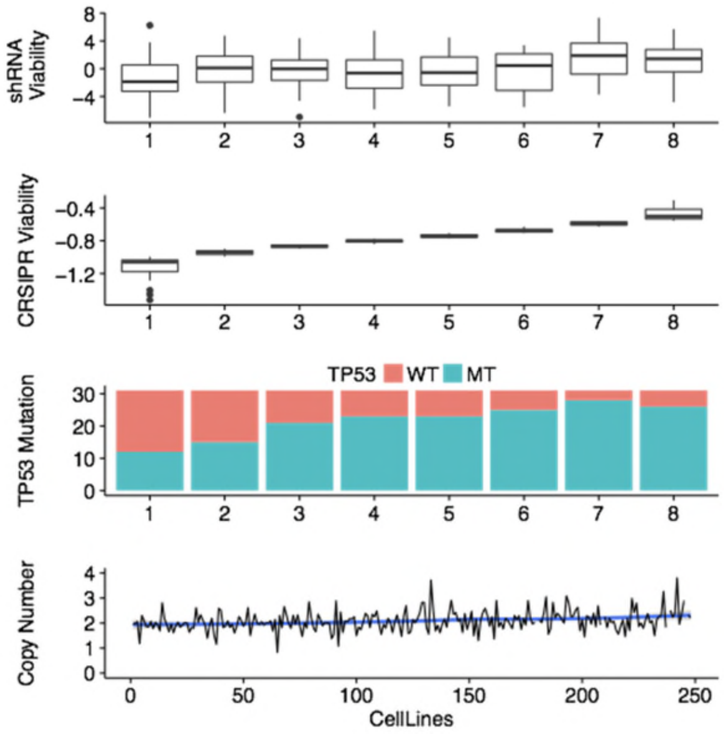

Supplementary Figure 11: Top CDE+ of p53: The distribution of *TAF8* (the top CDE+ gene of *p53*) post KO/KD viability scores in the CRISPR (top panel) screen as a function of *p53* mutation status. The cell lines were divided into 8 bins of equal size, which are ordered by their viability after the CRISPR-KO of *TAF8*. The fraction of *p53* mutant cell lines in each bin is plotted on the second panel. The distribution of *TAF8* post shRNA-KD viability scores is displayed on the third panel as a control. The fourth, lowest panel visualizes that the copy number of *TAF8* is about the same in the different bins. In the boxplots, the center line, box edges and whiskers denote the median, interquartile range and the rest of the distribution in respective order, except for points that were determined to be outliers using a method that is a function of the interquartile range, as done for standard box plots. N=248 cancer cell lines (total data points, biological replicates) used to derive the statistics in all the panels.

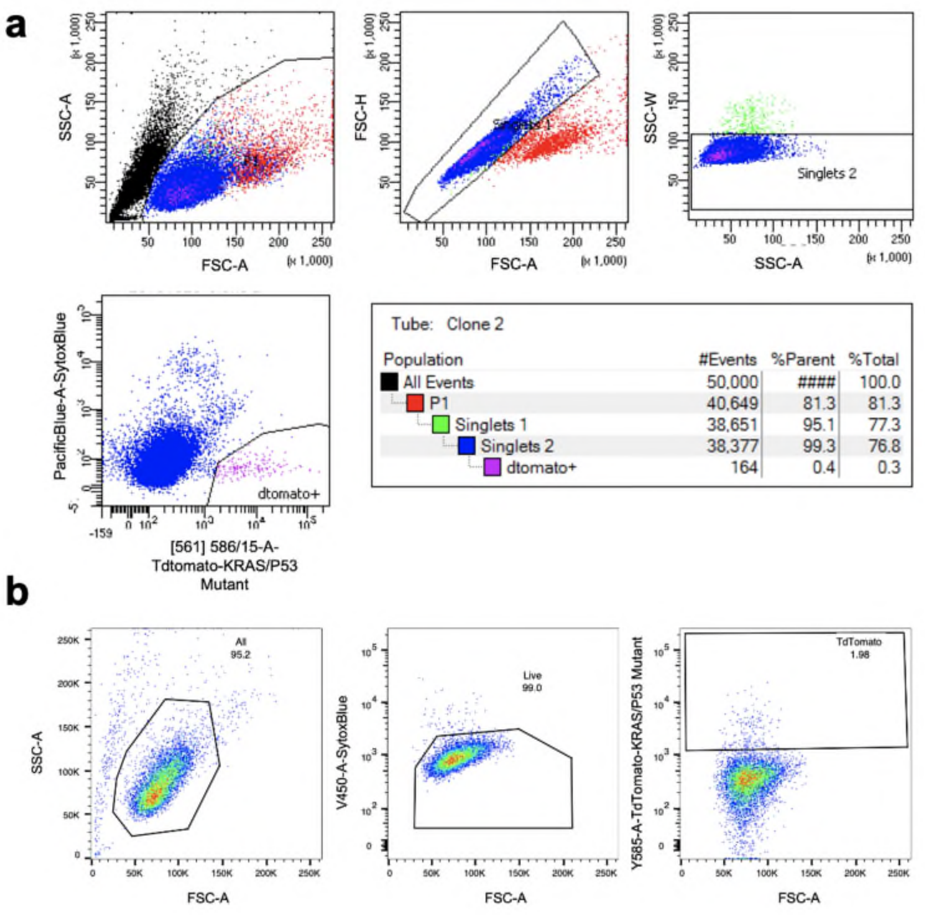

Supplementary Figure 12. **Schematic of FACS gating (a)** A representative example of gating used for sorting MOLM13 and RPE1 p53- and KRAS- mutant cells (Tomato+). **(b)** Gating strategy to analyze percentage of TdTomato positive cells expressing MIT-KRAS-G12D or MIT-p53-R248Q (bottom).

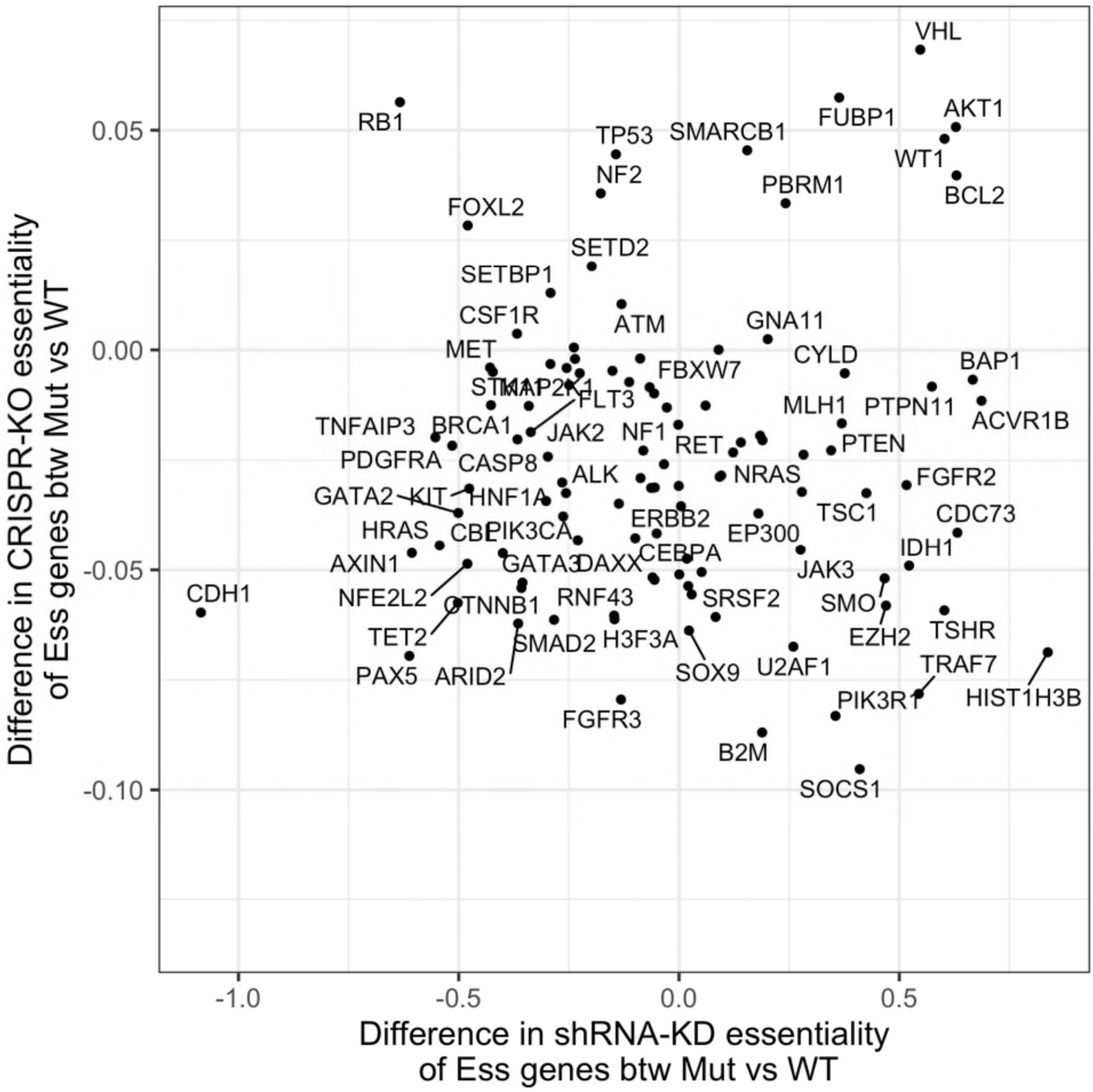

Supplementary Figure 13. **Potential Mutant selection susceptibility computed using the difference in essentiality score between driver Mut vs WT cell lines in CRISPR and shRNA screens using essential genes.** The mean difference in essentiality scores between driver Mut vs WT cell lines is computed using shRNA screens (X-axis) and CRISPR-Cas9 screen (Y-axis) using **(A)** essential genes. This is computed for each driver gene identified by Vogelstein et al. and labeled accordingly.

References

1. Behan, Fiona M., et al. "Prioritization of cancer therapeutic targets using CRISPR–Cas9 screens." Nature 568.7753 (2019): 511-516.
2. Brown, Kevin R., et al. "CRISPR screens are feasible in TP 53 wild- type cells." Molecular systems biology 15.8 (2019): e8679.
3. Hart, Traver, et al. "High-resolution CRISPR screens reveal fitness genes and genotype-specific cancer liabilities." Cell 163.6 (2015): 1515-1526.
4. Zimmermann, Michal, et al. "CRISPR screens identify genomic ribonucleotides as a source of PARP-trapping lesions." Nature 559.7713 (2018): 285-289.
5. Hart, Traver, et al. "Evaluation and design of genome-wide CRISPR/SpCas9 knockout screens." G3: Genes, Genomes, Genetics 7.8 (2017): 2719-2727.
6. Haapaniemi, Emma, et al. "CRISPR–Cas9 genome editing induces a p53-mediated DNA damage response." Nature medicine 24.7 (2018): 927-930.
7. Zhu, L. J., Holmes, B. R., Aronin, N. & Brodsky, M. H. CRISPRseek: a bioconductor package to identify target-specific guide RNAs for CRISPR-Cas9 genome-editing systems. PLoS One 9, e108424 (2014).
8. Doench, J. G. et al. Optimized sgRNA design to maximize activity and minimize off-target effects of CRISPR-Cas9. Nat. Biotechnol. 34, 184–191 (2016).
9. Yeo, N. C. et al. An enhanced CRISPR repressor for targeted mammalian gene regulation. Nat. Methods 15, 611–616 (2018).
10. Weinstein, J. N. et al. The Cancer Genome Atlas Pan-Cancer analysis project. Nature Genetics 45, 1113–1120 (2013).
11. Roe, J.-S. et al. Phosphorylation of von Hippel-Lindau protein by checkpoint kinase 2 regulates p53 transactivation. Cell Cycle 10, 3920–3928 (2011)
12. Luo, Ji, et al. "A genome-wide RNAi screen identifies multiple synthetic lethal interactions with the Ras oncogene." Cell 137.5 (2009): 835-848.
13. Martin, T. D. et al. A Role for Mitochondrial Translation in Promotion of Viability in K-Ras Mutant Cells. Cell Rep. 20, 427–438 (2017).
14. Ferrari, Samuele, et al. "Efficient gene editing of human long-term hematopoietic stem cells validated by clonal tracking." Nature biotechnology 38.11 (2020): 1298-1308.
15. Enache, Oana M., et al. "Cas9 activates the p53 pathway and selects for p53-inactivating

mutations." Nature genetics 52.7 (2020): 662-668.

16. Schiroli, Giulia, et al. "Precise gene editing preserves hematopoietic stem cell function following transient p53-mediated DNA damage response." Cell Stem Cell 24.4 (2019): 551-565.
