# Supplementary material for: A systematic genome-wide mapping of oncogenic mutation selection during CRISPR-Cas9 genome editing
Source: Nat Commun. 2021 Nov 11;12:6512. doi: 10.1038/s41467-021-26788-6 (PMC8586238; doi:10.1038/s41467-021-26788-6)
Supplement: Supplementary file 2 — Reporting Summary [file 41467_2021_26788_MOESM2_ESM.pdf]

nature portfolio

Corresponding author(s): Joo Sang Lee, Aniruddha J. Deshpande, Eytan Ruppin

Last updated by author(s): Sept 2, 2021

Reporting Summary

Nature Portfolio wishes to improve the reproducibility of the work that we publish. This form provides structure for consistency and transparency in reporting. For further information on Nature Portfolio policies, see our Editorial Policies and the Editorial Policy Checklist.

Statistics

For all statistical analyses, confirm that the following items are present in the figure legend, table legend, main text, or Methods section.

n/a	Confirmed	
☐	☒	The exact sample size (*n*) for each experimental group/condition, given as a discrete number and unit of measurement
☐	☒	A statement on whether measurements were taken from distinct samples or whether the same sample was measured repeatedly
☐	☒	The statistical test(s) used AND whether they are one- or two-sided<br>*Only common tests should be described solely by name; describe more complex techniques in the Methods section.*
☒	☐	A description of all covariates tested
☐	☒	A description of any assumptions or corrections, such as tests of normality and adjustment for multiple comparisons
☐	☒	A full description of the statistical parameters including central tendency (e.g. means) or other basic estimates (e.g. regression coefficient) AND variation (e.g. standard deviation) or associated estimates of uncertainty (e.g. confidence intervals)
☐	☒	For null hypothesis testing, the test statistic (e.g. *F*, *t*, *r*) with confidence intervals, effect sizes, degrees of freedom and *P* value noted<br>*Give P values as exact values whenever suitable.*
☒	☐	For Bayesian analysis, information on the choice of priors and Markov chain Monte Carlo settings
☒	☐	For hierarchical and complex designs, identification of the appropriate level for tests and full reporting of outcomes
☒	☐	Estimates of effect sizes (e.g. Cohen's *d*, Pearson's *r*), indicating how they were calculated

Our web collection on statistics for biologists contains articles on many of the points above.

Software and code

Policy information about availability of computer code

Data collection	Flow cytometry data was collected using the FACSDiva 8.0.1 software in the LSR Fortessa (BD Biosciences) was used for flow cytometry data collection. Next Generation Sequencing Data was collected using the MiSeq (Illumina).
Data analysis	Open Source softwares R 3.6.3 and Cytoscape 3.6.1 was used for analysis and visualizations. The required scripts to reproduce each step of results and figures can be accessed here: https://github.com/ruppinlab/crispr_risk.

For manuscripts utilizing custom algorithms or software that are central to the research but not yet described in published literature, software must be made available to editors and reviewers. We strongly encourage code deposition in a community repository (e.g. GitHub). See the Nature Portfolio guidelines for submitting code & software for further information.

Data

Policy information about availability of data

All manuscripts must include a data availability statement. This statement should provide the following information, where applicable:

- Accession codes, unique identifiers, or web links for publicly available datasets
- A description of any restrictions on data availability
- For clinical datasets or third party data, please ensure that the statement adheres to our policy

The required data from both the previously published and in-house screens, in their raw and processed form to reproduce each step of results and figures can be accessed here: https://github.com/ruppinlab/crispr_risk. Processed expression, mutation, copy number, CRISPR-Cas9 and shRNA pooled genetic screen data were derived from DepMap v19Q3 and can be found here (https://depmap.org/portal/). Copy number and mutation profile of all patient tumors available in TCGA were retrieved from the firehose pipeline (https://gdac.broadinstitute.org/). Functional and positional genesets were derived from MSigDB (https://www.gsea-msigdb.org/gsea/msigdb/). Cas9 activity in 1601 cell lines from DepMap and deep sequencing profiles of these Cas9-expressed vs matched cell lines were derived

from Enache et al. 2020 (Supplementary Tables).

Field-specific reporting

Please select the one below that is the best fit for your research. If you are not sure, read the appropriate sections before making your selection.

[x] Life sciences   [ ] Behavioural & social sciences   [ ] Ecological, evolutionary & environmental sciences

For a reference copy of the document with all sections, see nature.com/documents/nr-reporting-summary-flat.pdf

Life sciences study design

All studies must disclose on these points even when the disclosure is negative.

Sample size	Pooled CRISPR screens had 2 replicates per experimental condition and time point. All arrayed CRISPR screens had 3 replicates per condition and timepoint. No sample-size calculations were performed. The number of replicates used for genetic screens is standard in the field.
Data exclusions	All the data points in the publicly available data are used. For in-house screens, the exclusion criteria was pre-established for in-house and sgRNAs with greater than 20 reads are removed.
Replication	Pooled CRISPR screens had 2 replicates per experimental condition and time point. All arrayed CRISPR screens had 3 replicates per condition and time point. All attempts at replication were successful and experimental conditions were standardized with appropriate controls.
Randomization	To avoid cell line specific bias induced during randomization, we performed our genetic screens in isogenic cell lines and same experiemental conditions.
Blinding	This study is observational and not prospective, and thus didn't require blinding.

Reporting for specific materials, systems and methods

We require information from authors about some types of materials, experimental systems and methods used in many studies. Here, indicate whether each material, system or method listed is relevant to your study. If you are not sure if a list item applies to your research, read the appropriate section before selecting a response.

Materials & experimental systems

n/a	Involved in the study	
[ ]	[x]	Antibodies
[ ]	[x]	Eukaryotic cell lines
[x]	[ ]	Palaeontology and archaeology
[x]	[ ]	Animals and other organisms
[x]	[ ]	Human research participants
[x]	[ ]	Clinical data
[x]	[ ]	Dual use research of concern

Methods

n/a	Involved in the study	
[x]	[ ]	ChIP-seq
[ ]	[x]	Flow cytometry
[x]	[ ]	MRI-based neuroimaging

Antibodies

Antibodies used	Antibody used: APC anti-H2A.X-Phosphorylated (Ser139). Biolegend, San Diego, CA. Cat. # 613415. Lot # B309746. Clone # 2F3.
Validation	Validation: the antibody used was validated for its associated application by Biolegend and validated by literature citations available on the company website: https://www.biolegend.com/en-us/products/apc-anti-h2a-x-phosphorylated-ser139-antibody-13437

Eukaryotic cell lines

Policy information about cell lines

Cell line source(s)	hTERT RPE-1 cells were obtained from ATCC. MOLM13 cell lines are obtained from DSMZ.
Authentication	Short Tandem repeat profiling was perfomred to authenticate the identity of cell lines.
Mycoplasma contamination	Cell lines were not tested for mycoplasma contamination.
Commonly misidentified lines (See ICLAC register)	No commonly misidentified cell lines were used.

March 2021

Flow Cytometry

Plots

Confirm that:

☒ The axis labels state the marker and fluorochrome used (e.g. CD4-FITC).

☒ The axis scales are clearly visible. Include numbers along axes only for bottom left plot of group (a 'group' is an analysis of identical markers).

☒ All plots are contour plots with outliers or pseudocolor plots.

☒ A numerical value for number of cells or percentage (with statistics) is provided.

Methodology

Sample preparation	MOLM13 cells were harvested by centrifugation, washed once with PBS and resuspended in 400 ul PBS with 1X SytoxBlue for sorting. For competition assays and dye-dilution experiments, a cell culture sample was diluted with PBS with 1X SytoxBlue for flow cytometry analysis. Sampling volumes were determined to allow the acquisition of sufficient events for downstream analyses.
Instrument	BD LSR Fortessa and FACSAria III (BD Biosciences)
Software	FACSDiva 8.0.1 and Flowjo 9.
Cell population abundance	About 0.5%-5% of the total cells were Tdtomato positive. Purity of sorted population was checked using FACSAria III right after the sort and was above 95%.
Gating strategy	For sorting, scatter plots were generated using Area scatter of FSC and SSC. Then single cells were gated using FSC-A against FSC-H as wells as SSC-A against SSC-W. Single cells were gated for live TdTomato+ cells using PacificBlue-A (SytoxBlue) against Tdtomato-A to check the percentage of TdTomato+ cells. TdTomato+ population was separated by excluding the bottom 5% events appearing on/close to the boundary of positive and negative populations, considering controls without TdTomato. For analysis, scatter plots were generated using Area scatter of FSC and SSC. Cells were gated excluding debri. Cells were further gated for live populations using PacificBlue-A (SytoxBlue) against FSC-A (PacificBlue-ve). Live cells were plotted using SSC-A against Tdtomato-A to gate for the percentage of TdTomato+ events using a negative control.

☒ Tick this box to confirm that a figure exemplifying the gating strategy is provided in the Supplementary Information.
