# Supplementary material for: A systematic genome-wide mapping of oncogenic mutation selection during CRISPR-Cas9 genome editing
Source: Nat Commun. 2021 Nov 11;12:6512. doi: 10.1038/s41467-021-26788-6 (PMC8586238; doi:10.1038/s41467-021-26788-6)
Supplement: Supplementary file 4 — Description of Additional Supplementary Files [file 41467_2021_26788_MOESM4_ESM.pdf]

Title: Supplementary Data 1.
Description: *List of cell lines and genes present in both AVANA and Achilles, which has been used in this study.*

Title: Supplementary Data 2.
Description: CDE+ and CDE- genes and their enriched pathways

Title: Supplementary Data 3.
Description: *Chromosomal common fragile sites (CFSs) enrichment analysis*

Title: Supplementary Data 4:
Description: sgRNA sequence in competition assay

Title: Supplementary Data 5.
Description: *Fisher test significance of candidates from Vogelstein et al. which are mutated in at least 10 samples*

Title: Supplementary Data 6.
Description: *List of overlapping CDE+ genes across p53 and KRAS*

Title: Supplementary Data 7.
Description: *CRISPR-KO and CRISPRi screens of CDE genes in KRAS isogenic MOLM13 cell lines*

Title: Supplementary Data 8.
Description: *Competition assay of KRAS isogenic MOLM13 cells under CRISPR-KO of CDE genes*

Title: Supplementary Data 9.
Description: *Primer Sequences used in the study*
